# Effectiveness and safety of techniques for cervical spine immobilization in mountain rescue

**DOI:** 10.1186/s13049-025-01530-z

**Published:** 2026-01-16

**Authors:** Richard F. Kraus, Maximilian L. Knipfer, Matthias Jacob, Baerbel Kieninger, Jasmine Alikhani, Parham Heydarzadeh Ghamsary, Lukas Reinker, Ina Adler, Sebastian Dendorfer, Martin Kieninger

**Affiliations:** 1 Department of Anesthesiology, University Hospital Regensburg, Franz‐Josef‐Strauss‐Allee 11, 93053 Regensburg, Germany; 2 Laboratory for Biomechanics, Center for Biomedical Engineering, Ostbayerische Technische Hochschule (OTH) Regensburg, Seybothstraße 2, Regensburg, 93053 Germany; 3 Bavarian Mountain Rescue Service, Am Sportpark 6, 83646 Bad Tölz, Germany; 4 Department of Infection Prevention and Infectious Diseases, University Hospital Regensburg, Franz‐Josef‐Strauss‐Allee 11, 93053 Regensburg, Germany; 5 Department of Anesthesiology, Barmherzige Brüder St.-Elisabeth-Hospital Straubing, St.-Elisabeth-Straße 23, 94315 Straubing, Germany; 6 BExMed Scientific Committee (German Society of Mountain and Expedition Medicine), Manzostraße 72, Munich, 80997 Germany

**Keywords:** Mountain rescue, Orthosis, Cervical spine, Immobilization, Vacuum mattress

## Abstract

**Background:**

Cervical spine injuries in alpine sports require immediate immobilization at the site of the accident to avoid possible secondary damage caused by transportation. Using special sensor technology, this study investigated whether a cervical spine orthosis (cervical collar, Stifneck collar (Laerdal Medical GmbH, Puchheim, Germany)) provides greater stability than a vacuum mattress alone.

**Methods:**

Using one male test person, we simulated transporting a patient with a spinal injury in steep alpine terrain. A wireless motion capture system (Xsens Technologies, Movella™ Inc., Henderson, USA) was used to record motion in three-dimensional space within a standardized environment. All tests were performed on a set course by the Bavarian Mountain Rescue Service. The test person lay on a mountain rescue stretcher and was immobilized with a vacuum mattress, either with or without a cervical orthosis. The axes of cervical spine movements were analyzed separately.

**Results:**

There were no significant differences between immobilization with and without a cervical orthosis with regard to lateral flexion (max. 3.7° compared to 3.0°) in the frontal plane and maximum excursion in flexion (max. 1.6° compared to 2.8°) or extension (max. -1.6° compared to -1.7°). There was significantly greater rotation movement around the craniocaudal axis without an orthosis (max. 2.4° compared to 1.3°).

**Conclusion:**

During mountain rescues, the cervical spine can be immobilized without a rigid cervical spine orthosis. Future research should explore the fundamental benefits of cervical spine immobilization, while the findings of this work contribute to the safe care of patients by avoiding the disadvantages associated with rigid cervical orthoses.

**Supplementary Information:**

The online version contains supplementary material available at 10.1186/s13049-025-01530-z.

## Introduction

The wide variety of alpine mountain sports, each with its own biomechanical loads, results in many different injury patterns [1–5]. Providing medical care in an alpine environment often poses a big challenge to the mountain rescue teams. Since alpine sports are often practiced in difficult-to-access terrain, rescue times are correspondingly long, in extreme cases up to several days [6, 7].

Injuries to the cervical spine are not the most common in alpine sports, but they require immediate immobilization at the site of the accident to avoid possible secondary damage caused by transportation [2]. Injuries to the bones of the cervical spine can lead to spinal cord damage and must be avoided at all costs [1, 6].

A systematic approach to trauma care has been established through the introduction of standardized treatment concepts such as the Advanced Trauma Life Support (ATLS®) or Prehospital Trauma Life Support (PHTLS®) [8]. Decision aids, such as the Canadian C-Spine Rule or the criteria of the National Emergency X-Radiography Utilization Study (NEXUS), have proven useful in assessing the need for cervical spine immobilization [9, 10].

In 2001, Stiell et al. published a study in Canada that investigated the subsequent heterogeneous and ineffective ordering of X-ray diagnostics of the cervical spine [11]. Since then, the use of imaging diagnostics for the cervical spine in emergency rooms has evolved for prehospital use and now serves as an additional decision aid for determining the necessity of cervical spine immobilization.

However, recommendations for changes to the guidelines were published in 2022 (e.g. by Cowley et al.) to reflect the evolving practice of prehospital spine immobilization. New guidelines (even if not “formally” validated but at least based on the latest evidence) have been published with the aim of reducing the potential for iatrogenic harm and promoting a patient-centered approach [12].

Nevertheless, up until now, if the C-Spine Rule or the NEXUS criteria indicate the need for prehospital cervical spine immobilization, the mountain rescue team will perform it using a vacuum mattress and a cervical spine orthosis.

However, there is a lack of studies on prehospital care, especially with regard to mountain rescues, that take the unique alpine conditions into account. Transporting injured patients over difficult terrain increases the force on their cervical spine. It must be investigated whether the currently used techniques in the form of vacuum mattresses and rigid cervical spine orthoses comply with these special requirements. The rigid cervical spine orthosis, which has been the standard for decades, is increasingly disputed. In the literature, there is growing doubt about the usefulness of such orthoses due to their negative side effects. When applying a cervical spine orthosis, particular attention must be paid to the increase in intracranial pressure [13]. Also, skin lesions [14] may be a more pressing concern than previously assumed due to the long transportation times in mountain rescues.

The potential harm caused by cervical orthoses is particularly pertinent in scenarios involving prolonged prehospital periods, exposure to low or high temperatures, or challenging terrain, such as during mountain rescues, where the associated risks may be amplified (recently reviewed by Pandor et al. [15]).

Therefore, the objective of our study was to investigate the effectiveness of the immobilization techniques currently used in mountain rescues. We analyzed whether the use of a vacuum mattress and a rigid cervical spine orthosis results in better immobilization of the cervical spine than the use of a vacuum mattress alone.

## Materials and methods

### Human ethics and consent to participate declaration

After a positive vote of the Ethics Committee of the University of Regensburg (file number 20-1661-101), the study was conducted with one voluntary test person, who gave informed consent according to the Declaration of Helsinki.

### Mountain rescue materials for the immobilization and rescue of patients

**Fig. 1:**
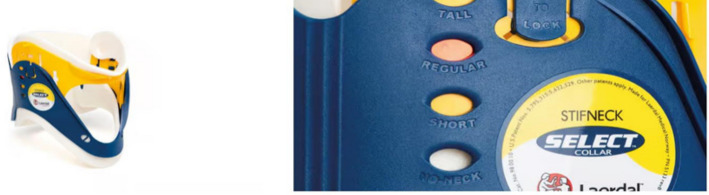
The cervical spine is often immobilized using rigid cervical orthoses (currently often in addition to a vacuum mattress). The model most commonly used by the Bavarian Mountain Rescue Service was also used in this study: Stifneck Select with adjustment options (Laerdal Medical GmbH) [16]

The mountain rescue service has a wide range of equipment available for the care of accident victims and their subsequent transportation. In order to realistically simulate a mountain rescue operation, our study used the same materials as the Bavarian Mountain Rescue Service. These materials partly include medical products for general prehospital care, such as the Stifneck® Select™ cervical spine orthosis from Laerdal (Laerdal Medical GmbH, Puchheim, Germany) (see Fig. 1). This orthosis consists of polyethylene coated with 2 mm of foam and is closed with a hook-and-loop fastener.

The cervical spine was stabilized by supporting the occiput and mandible against the sternum, clavicle, trapezius muscle, and upper back. To achieve optimal immobilization, the product had to be adapted to the patient [14]. For this purpose, four different sizes were available, which were selected based on the patient’s chin-to-shoulder distance according to the manufacturer’s instructions [17].

**Fig. 2:**
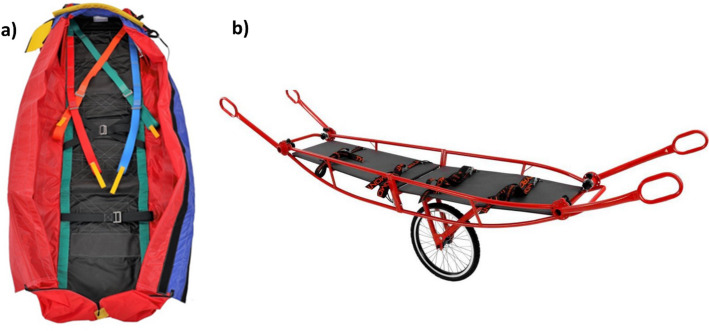
Immobilization and transport tools used in this study. **a** Helicopter rescue bag model “Bayern” (TYROMONT Alpin Technik GmbH) [19]. **b** Steelight mountain stretcher (TYROMONT Alpin Technik GmbH) [20]

Further immobilization was carried out using an “80 – Bayern” type vacuum mattress from Tyromont (TYROMONT Alpin Technik GmbH, Thaur, Austria). The extensive belt system enables the mattress to be precisely adapted and fixed to the respective injured body region of the patient [18]. This product is designed for use in combination with the helicopter rescue bag model “Bayern” from Tyromont (see Fig. 2a). The design of the bag ensures that the vacuum mattress stays in place without the risk of dislocation, but it does not provide actual immobilization [19]. The test person was transported with a Steelight mountain stretcher from Tyromont (see Fig. 2b, [20]). The four adjustable handles enable the mountain rescue personnel to handle it safely, even in difficult terrain.

### Motion measurement with the Xsens Link system

**Fig. 3:**
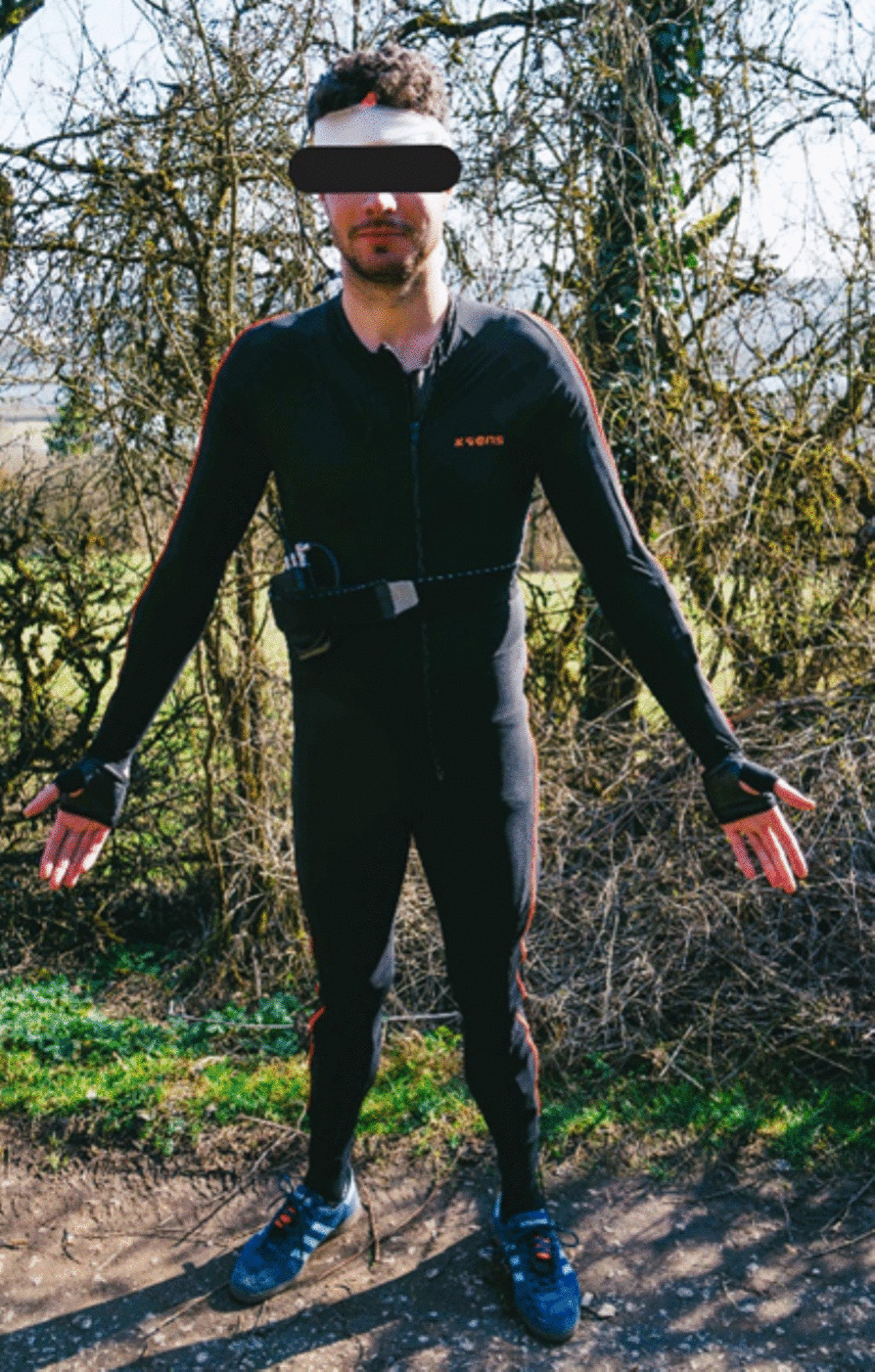
A volunteer test person wore the measurement suit, which incorporated 17 single sensors for recording the movement of the cervical spine

In this study, movements of the cervical spine immobilized during a simulated mountain rescue operation were recorded using various methods, for instance with the Xsens Motion Capture System from Movella™ (Movella™ Inc., Henderson, USA). In the Xsens Link configuration used for this study, the test person wore a Lycra suit (see Fig. 3) containing 17 incorporated inertial measurement units (see Table S 1 in the supplement) that recorded acceleration, position, and inclination via a 3D accelerometer, 3D gyroscope, 3D magnetometer, and barometer. Data were recorded at a frequency of 240 Hertz (Hz) [21].

### Measurement route for a realistic simulation of a rescue operation

**Fig. 4:**
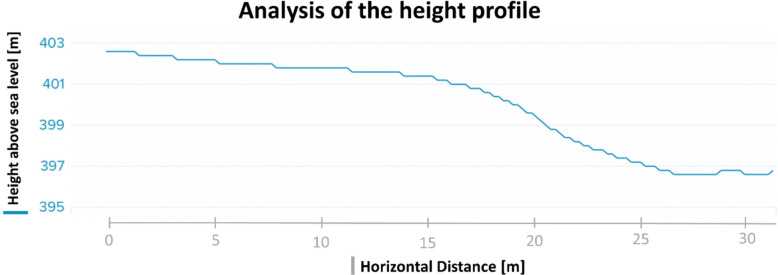
Height profile of the test route, recorded with a Suunto 5 GPS watch (Suunto Oy, Vantaa, Finland). The x-axis shows the distance traveled on the transport route [m], the y-axis shows the change in altitude [m]

To achieve a realistic simulation of the conditions during a mountain rescue operation, it was essential to choose a suitable terrain in which to conduct the measurements. Therefore, the test person was transported along a 31-m sloping path (Fig. 4), which involved passing over a gravel path, a forest path, and a meadow.

**Fig. 5:**
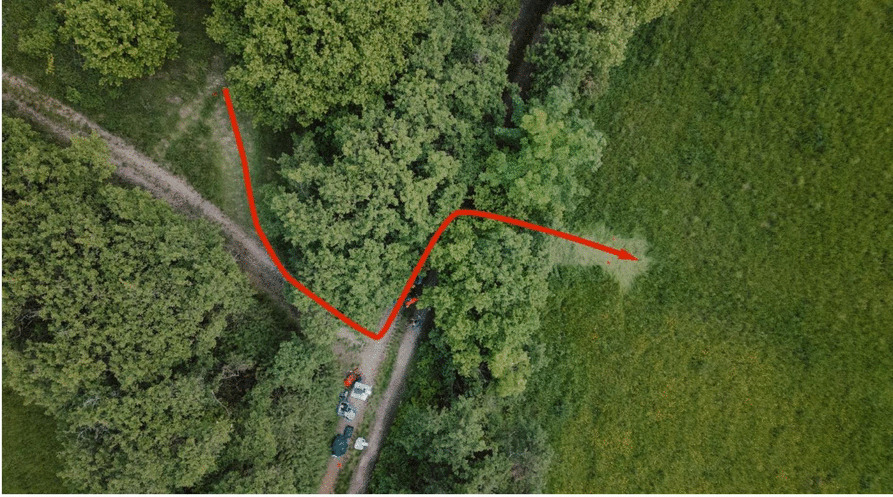
General view of the measurement route (bird's-eye view image taken with a drone). The red line indicates the route taken during the measurements with the mountain stretcher

**Fig. 6:**
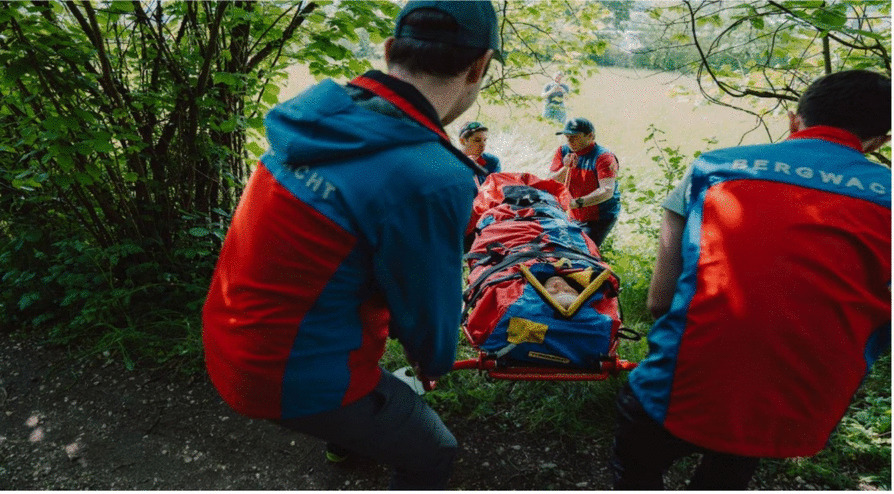
Transportation of the test person by members of the mountain rescue service

The route contained a 90° bend (see Fig. 5), and the patient had to be carried to a lower-lying grass area by being lifted over a hedge approximately 1.7 m high (see Fig. 6 and video 1 in the supplement). Furthermore, a square piece of timber with a side length of 4.5 cm had to be passed over.

### Measurement of the forces acting on the spinal cord during patient transportation as part of a simulated mountain rescue operation

Before the start of the measurement series, the Xsens measurement system had to be calibrated. For this purpose, the anthropometric parameters of the test person were stored in the software (see Table S 2 in the supplement). After the sensors had been applied, the test person had to remain in a resting position for 2–3 s (s), then walk approximately 10 m and subsequently return to the initial position. During this time, the system calibrated and saved the position of the sensors in relation to each other and the body segments of the test person.

As our study focused on patient transportation, measurements only began once the test person was placed on the mountain stretcher and immobilized using the respective techniques. A 30-s calibration run was carried out before each measurement to determine the resting position, after which the transportation started. Four active, trained members of the Bavarian Mountain Rescue Service carried the mountain stretcher along the specified course (see video 2 in the supplement).

The movements recorded in the process were stored locally by the measurement system in the “on-body recording” mode. After completing the measurement route, the recording was finished, and the test person on the mountain stretcher was taken back to the starting point. The measurements were repeated 30 times: 15 runs with the test person immobilized using a vacuum mattress, and further 15 runs with the test person immobilized using a vacuum mattress and a cervical spine orthosis.

### Processing of the measurements

**Fig. 7:**
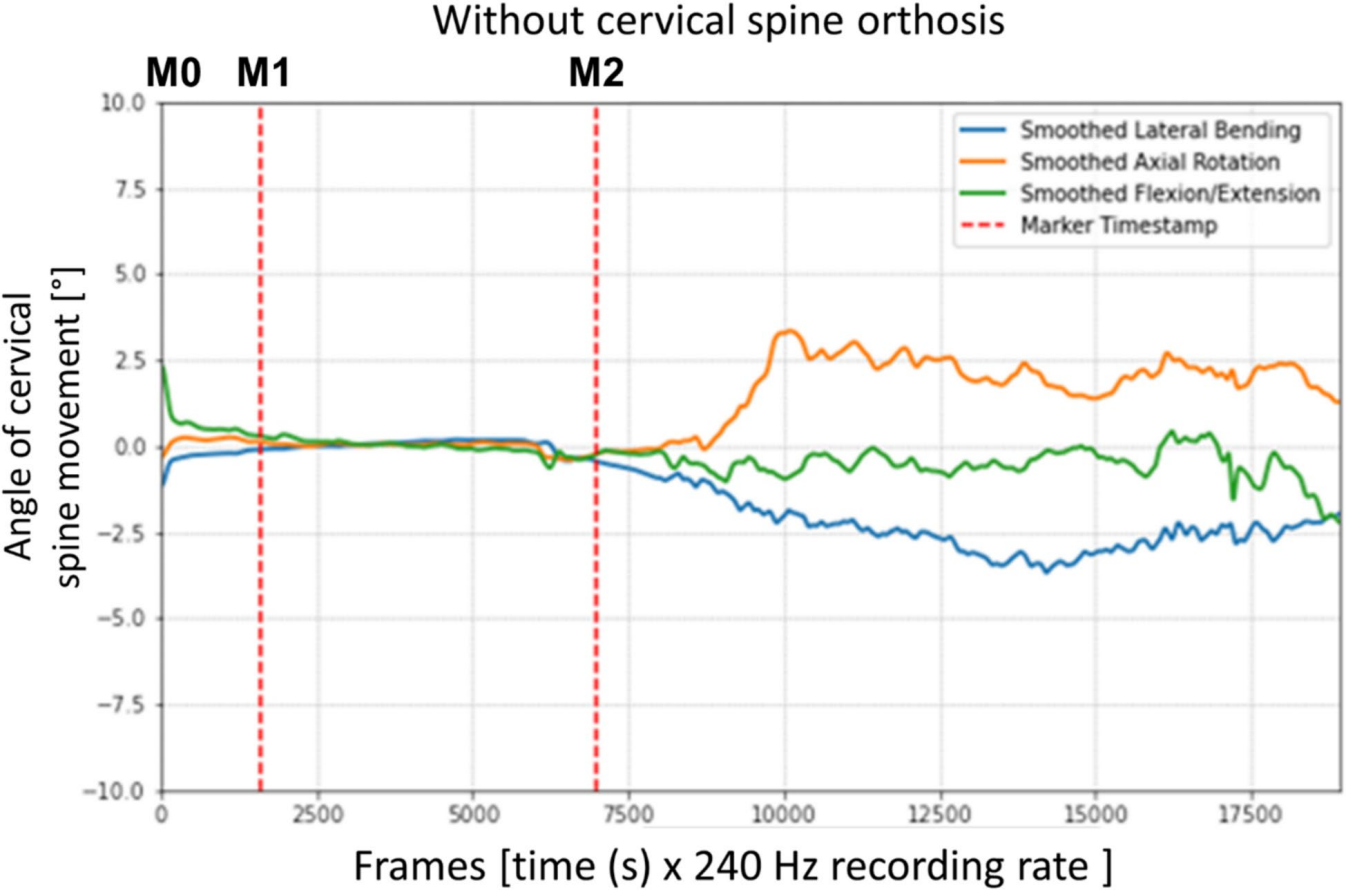
Graphical representation of a test run without a cervical spine orthosis. The x-axis shows the measurement times (frames), recorded at a frequency of 240 per second [Hz], while the y-axis shows the angle changes [°]

The measured data had to be processed in several steps before a statistical evaluation could be conducted (see Figure S2 in the supplement). The values collected and stored locally in the measurement system had to be transferred to the MVN Analyze Software (Version 2024 2.0, Movella™ Inc., Henderson, USA). Segment orientations and relative joint movements were estimated by combining the inertial sensor data with the test person's anthropometric parameters. The subsequently generated dataset was exported to Excel (Version 2402, Microsoft Corporation, Redmond, USA). For each measurement run, a separate file was created containing the recorded measurements of all 17 sensors. The datasets were reduced to include only the cervical spine data relevant to our study. The range of motion of the cervical spine was measured by the degree to which the sensor on the head deflected in relation to the sensor on the sternum, which was defined in the system as “ergonomic joint angle Head_T8”. The captured movements were recorded as Euler angles, with the angles described in ZXY format: Z as the craniocaudal axis, X as the sagittal axis, and Y as the horizontal axis. The data were further processed using a specifically developed Python script (Version 3.12.1, see Python Script in the supplement). The first step was to convert the Excel files into TSV format. The relevant time events in the measuring sequence were then defined by setting time markers for each measurement run. The marker M0 marked the beginning of the experiment; M1 marked the beginning of the 30-s zero adjument, which ended at M2 and, at the same time, marked the start of the transport of the test person (see Fig. 7). These time markers were adjusted according to the time course of the respective run. Any inaccuracies in the measurements taken by the Xsens capture system that fell below its tolerance level were smoothed out. For this purpose, the plots output by the Python script for the graphical representation of the measurements were reviewed, and each axis was analyzed individually. For the processing with a Gaussian smoothing filter, a kernel size of 30 was set for flexion/extension and lateral flexion, and a kernel size of 40 was set for rotation (see Figure S 1 in the supplement). Finally, a conversion back to Excel files was carried out for each of the 30 runs, whereby the minimum, maximum, and mean values with standard deviation, median as well as 25% and 75% quartile were calculated for the three axes of movement. In addition, the size of the swept angle range was calculated as follows: the maximum value minus the maximum value in the opposite direction.

### Statistics

For the guidance and selection of the statistical test procedures, the method consulting homepage of the University of Zurich was used [22]. A detailed description of the statistical methods can be found in the supplement (see section S1).

## Results

As part of the 30 test runs, which had an average duration of 47 s, the first 15 measurements were carried out without a cervical spine orthosis. After the application of the cervical spine orthosis and recalibration, 15 further measurements followed. Figure 7 shows the graphical course of a representative measurement run without a cervical spine orthosis. The three curves (blue: lateral flexion, orange: rotation, green: flexion/extension) represent the recorded measuring values. The angles occurred (°) are plotted on the y-axis as a function of time (frames). The frames displayed on the x-axis represent the individual measuring points recorded at 240 Hz (see Sect. "Processing of the measurements"). The red, dashed vertical lines show the range within which the calibration to determine the resting position was performed (M0 + M1). Movements that occurred before this time are due to the preparations for the calibration and were not included in the evaluation.

In comparison to Fig. 7, it becomes clear that the individual adjustment of the time markers, as described in the material and methods section, was made to optimally limit calibration times.

### Results of the analysis of lateral flexion in the frontal plane

Table 1 summarizes the maximum values, the mean deviation from zero, and the size of the swept angle range calculated for the respective test runs with and without a cervical spine orthosis. For the maximum and mean values, no distinction was made between lateral flexion to the left and to the right due to the symmetry of the head (assuming that the head is symmetrical and that markers were placed precisely at the center of the head). Therefore, the absolute values were used. In contrast, the values that occurred in the respective directions of movement (real values) were taken into account when calculating the range. When looking at the parameters for lateral flexion in the frontal plane, there were no significant differences between the runs without and with a cervical spine orthosis.

**Table 1:** Results of lateral flexion degrees [°] of the cervical spine in the frontal plane

Lateral flexion	Maximum value*	Mean value*	Range
Group 0	Median: 3.0°	Median: 1.1°	Median: 3.2°
(without a cervical spine orthosis)	IQR: 1.9°	IQR: 1.6°	IQR: 1.0°
Group 1	Median: 3.7°	Median: 1.6°	Median: 4.8°
(with a cervical spine orthosis)	IQR: 3.3°	IQR: 2.5°	IQR: 3.4°
Comparison (*p*-value)	MWU test: *p* = 0.074	MWU test: *p* = 0.116	MWU test: *p* = 0.074

Parameters marked with an *: calculation with absolute values

### Results of the analysis of rotation around the craniocaudal axis

The rotation values were analyzed in the same way as those of lateral flexion. The maximum and mean values were evaluated on the basis of the absolute values, whereas the real values were used for the range (see Table 2). When looking at the rotation around the craniocaudal axis, significantly bigger movements were seen during the runs without a cervical spine orthosis than during runs with a cervical spine orthosis.

**Table 2:** Results of rotation degrees [°] around the craniocaudal axis

Rotation	Maximum value*	Mean value*	Range
Group 0	Median: 2.4°	Median: 0.7°	Median: 3.7°
(without a cervical spine orthosis)	IQR: 1.2°	IQR: 0.7°	IQR: 2.1°
Group 1	Median: 1.3°	Median: 0.5°	Median: 1.8°
(with a cervical spine orthosis)	IQR: 0.4°	IQR: 0.2°	IQR: 0.8°
Comparison (*p*-value)	MWU test: *p* < 0.001	MWU test: *p* < 0.001	MWU test: *p* < 0.01
Parameters marked with an *: calculation with absolute values			

### Results of the analysis of flexion and extension in the sagittal plane

In order to capture the actually occurred directions of movement, the angles of flexion and extension movements were analyzed using the real values measured. Positive angles represent flexion movements, while negative angles represent extension movements. When looking at the flexion and extension in the sagittal plane, a significant difference in the range values was found between the runs with and without a cervical spine orthosis, while no significant difference could be detected between the minimum, maximum, and mean values (see Table 3).

**Table 3:** Results of flexion/extension degrees [°] of the cervical spine in the sagittal plane

Flexion/Extension	Extension	Flexion	Mean value	Range
Group 0	Median: −1.6°	Median: 2.8°	Median: −0.2°	Median: 4.1°
(without a cervical spine orthosis)	IQR: 2.3°	IQR: 2.9°	IQR: 1.9°	IQR: 1.7°
Group 1	Median: −1.7°	Median: 1.6°	Median: −0.5°	Median: 2.9°
(with a cervical spine orthosis)	IQR: 1.1°	IQR: 1.6°	IQR: 1.0°	IQR: 1.4°
Comparison (*p*-value)	MWU test: *p* = 0.806	MWU test: *p* = 0.137	MWU test: *p* = 0.116	MWU test: *p* = 0.023

All parameters were calculated using the real values

## Discussion

### Establishing a suitable test scenario and executing and optimizing measurements

A measurement system that has already been proven in several studies on cervical spine immobilization at the University Hospital of Regensburg [23, 24] was also used in this study to meet the special requirements of mountain rescue operations. The recorded values were processed according to the methods described under Sect. "Processing of the measurements" in order to be able to make reliable and reproducible statements. A detailed description of this approach can be found in the supplement (see section S2).

### Discussion of the observed results

The analysis showed that the movements in all axes were, at most, in the single-digit degree range. The results of the lateral flexion test showed that using a cervical spine orthosis in addition to other immobilizing methods did not result in any statistically significant advantages in the frontal plane. Conversely, use of a cervical spine orthosis resulted in even higher values (3.7° at the median compared to 3.0°). This finding could be due to the design of the used orthoses described in 2.2, which only offer limited lateral stabilization in the frontal plane due to the ventral and dorsal support. In both reference groups, the vacuum mattress was thus the main basis of immobilization. Analysis of the rotational movement around the craniocaudal axis showed significant differences between immobilization using a vacuum mattress alone versus a combination of a vacuum mattress and cervical spine orthosis. However, even when using a vacuum mattress alone, the maximum value was only increased by 1.1° at the median (2.4° compared to 1.3°). The mean value was 0.2° higher at the median (0.7° compared to 0.5°). The overall low angles in the low single-digit degree range are proof of good stabilization, even when using a vacuum mattress alone. When looking at the sagittal plane, flexion and extension movements must be evaluated separately. With regard to the extension movements, almost identical values were shown at the median (−1.6° compared to −1.7°). The median values of the flexion movement were lower with than without a cervical spine orthosis (1.6° compared to 2.8°), but this difference was not significant. The range between flexion and extension showed a significantly lower range of motion when using a cervical spine orthosis (2.9° compared to 4.1°).

A final evaluation of the results is not yet possible due to a lack of studies on the extent of movement that may cause damage to the spinal cord in cases of existing cervical spine injuries. However, since all values of all movement axes are in the low single-digit range, it seems unlikely that slight movements during cervical spine immobilization would have a relevant impact on the occurrence of secondary damage.

In summary, our study emphasizes that once the indication for cervical spine immobilization is established—regardless of the underlying reasons, which were not the focus of our investigation—, the cervical orthosis can be removed after the patient has been positioned on a vacuum mattress on a mountain rescue stretcher. Figure S 3 in the supplement summarizes the findings of our investigation on immobilization and those of other studies. It also shows a possible procedure for providing care to injured persons that takes cervical spine immobilization into account.

### Discussion in the context of related studies on cervical spine immobilization

The current procedure in mountain rescue is to apply a cervical spine orthosis as soon as possible after arrival at the emergency site and before positioning the patient on the vacuum mattress. The cervical spine orthosis then remains on the patient throughout the entire transportation, and is removed in the emergency room depending on the assessment of the hospital staff. A possible advantage of this procedure, namely the application of the cervical spine orthosis during the repositioning from the ground to the stretcher, has been proven in several scientific studies [23].

However, this procedure is increasingly criticized. Kieninger et al. found that with proper use of a vacuum mattress, patients can be transported in an ambulance without a rigid cervical spine orthosis [24]. Moreover, Uzun et al. were not able to demonstrate an additional advantage of using a rigid cervical spine orthosis [25]. So far, the special requirements of mountain rescue have only been investigated in special studies such as by Grenier et al. on skiing accidents, wherebyvacuum mattresses provided good stabilization, even without using a cervical spine orthosis [26].

In summary, our results are consistent with those of existing literature. Taking into account the already proven negative effects of rigid cervical collars [15], which worsen with increased wearing time, we can no longer recommend the use of rigid cervical collars during long mountain rescue transports with the mountain stretcher. We therefore recommend revising current guidelines for mountain rescue operations, with the findings of this study (maybe by taking into account our possible proposal shown in Figure S 3 and new developments in immobilization techniques discussed in section S3 in the supplement) being given due consideration.

### Limitations

A detailed discussion of the limitations of this study can be found in section S4 in the supplement.

## Conclusion

The prehospital care of patients, particularly in the challenging environment of mountain rescue, requires the continuous evolution of strategies and procedures to ensure safety and efficiency. This study took the particular difficulties in mountain rescues into account and demonstrated that sufficient immobilization is possible without the use of rigid cervical spine orthoses, even in difficult mountain rescue conditions.

Our study emphasizes that, once cervical spine immobilization is deemed necessary, cervical orthoses may be removed after immobilization of the patient on a vacuum mattress, even in mountainous terrain. Figure S 3 in the supplement summarizes immobilization findings from our investigation and other studies and outlines a potential care procedure for injured persons, considering cervical spine immobilization in mountain rescue.

Future studies should address the fundamental benefits of cervical spine immobilization. Until then, the results of the present study will serve to ensure safe patient care by avoiding the disadvantages associated with rigid cervical spine orthoses.

## Supplementary Information

Supplementary Material 1. Video 1.

Supplementary Material 2. Video 2.

Supplementary Material 3. Python script for data processing.

Supplementary Material 4. Original Data of the measurements.

Supplementary Material 5. Figure S1: Comparative plots without (a) and with (b) smoothing. The x-axis shows the measurement times (frames), recorded at a frequency of 240 per second [Hz], while the y-axis shows the angle changes [°]. Figure S2: Schematic overview of the evaluation steps. Figure S3: Proposal for a possible procedure for patient care with regard to cervical spine immobilization in mountain rescue based on the findings of this study. Table S1: Placement of the sensors [21]. Table S2: Anthropometric data of the test subject [21].

Supplementary Material 6. Section S1: Statistical Methods. Section S2: Evaluation of the measured results. Section S3: Development of Immobilization Materials. Section S4: Limitations.

## Data Availability

Data are available due to privacy/ethical restrictions. Original data of the measurements can be found in the supplement.
