# Supplementary material for: Effectiveness and safety of techniques for cervical spine immobilization in mountain rescue
Source: Scand J Trauma Resusc Emerg Med. 2026 Jan 16;34:12. doi: 10.1186/s13049-025-01530-z (PMC12849128; doi:10.1186/s13049-025-01530-z)
Supplement: Supplementary file 3 — Supplementary Material 3. Python script for data processing. [file 13049_2025_1530_MOESM3_ESM.pdf]

Python script for data processing

```
import os

import pandas as pd

import matplotlib.pyplot as plt

import argparse

import sys

from scipy.ndimage import gaussian_filter1d

import shutil

from colorama import Fore, Style, init


# Initialize colorama for cross-platform support

init(autoreset=True)


# Options for Excel-to-TSV conversion

OPTIONS = {

"timestamp_sheet": "Markers", # Name of the sheet containing timestamps

"data_sheet": "Ergonomic Joint Angles ZXY", # Name of the sheet containing data

"column_names": [

"Frame",

"T8_Head Lateral Bending",

"T8_Head Axial Bending",

"T8_Head Flexion/Extension"

],

}


# Function to extract timestamps from the Excel file

def timestamps_from_file(file) -> pd.DataFrame:

"""

Extracts timestamp markers from an Excel file. Warns if the cells are empty.


Parameters:

file (str): Path to the Excel file.


Returns:

DataFrame: DataFrame containing marker IDs and timestamps.

"""

# Read the specified sheet

df = pd.read_excel(file, sheet_name=OPTIONS["timestamp_sheet"])

# Extract timestamp values

timestamps = df["Frame"].values.tolist()

# Check if all cells in the "Frame" column are empty

if not any(timestamps): # Equivalent to checking if all cells are empty

print(f"{Fore.YELLOW}WARNING: No time markers found in '{file}'. Please fill the corresponding TSV file manually.{Style.RESET_ALL}")

# Create marker IDs

marker_ids = [f"M{i}" for i in range(len(timestamps))]

# Return as a DataFrame

return pd.DataFrame({"marker_id": marker_ids, "timestamp": timestamps})


# Function to extract data from the Excel file

def data_from_file(file) -> pd.DataFrame:

"""

Extracts motion data from an Excel file.


Parameters:

file (str): Path to the Excel file.


Returns:

DataFrame: DataFrame containing motion data with selected columns.

"""

df = pd.read_excel(file, sheet_name=OPTIONS["data_sheet"], usecols=OPTIONS["column_names"])

return df


# Function to transform raw Excel files into TSV format

def excel_to_tsv(input_folder, output_folder):

"""

Converts raw Excel files to TSV format for further processing.


Parameters:

input_folder (str): Directory containing raw Excel files.

output_folder (str): Directory where TSV files will be saved.


Saves:

- Data TSV files: Contain motion data.

- Marker TSV files: Contain timestamp markers.

"""

ensure_directory_exists(output_folder)


# List and sort Excel files in the input folder

files = os.listdir(input_folder)

files = [f for f in files if f.endswith(".xlsx")]

files = [os.path.join(input_folder, f) for f in files]

files.sort()


for file in files:

file_no_ext = os.path.splitext(os.path.basename(file))[0]

print(f"Processing: {file_no_ext}")


# Extract and save timestamp markers

timestamps = timestamps_from_file(file)

with open(os.path.join(output_folder, f"{file_no_ext}_markers.tsv"), "w", encoding="utf-8") as f:

timestamps.to_csv(f, index=False, header=True, sep="\t")


# Extract and save motion data

data = data_from_file(file)

with open(os.path.join(output_folder, f"{file_no_ext}.tsv"), "w", encoding="utf-8") as f:

data.to_csv(f, index=False, header=True, sep="\t")


def check_environment(expected_env='rescue'):

"""

Check if the script is running in the expected Conda environment.


Parameters:

expected_env (str): The name of the expected Conda environment.


Raises:

SystemExit: If the script is not running in the expected environment.

"""

# Get the current Conda environment name from environment variables

current_env = os.environ.get('CONDA_DEFAULT_ENV', None)

if current_env != expected_env:

print(f"ERROR: This script is expected to run in the '{expected_env}' Conda environment.")

print(f"Current environment: {current_env if current_env else 'None'}")
```

```
print("Please activate the correct environment and rerun the script.")

sys.exit(1)




def ensure_directory_exists(directory):

"""

Ensure that a directory exists. If it doesn't, create it.

Parameters:

directory (str): Path to the directory to check or create.

Raises:

Exception: If directory creation fails.

"""

try:

os.makedirs(directory, exist_ok=True)

print(f"Checked/created directory: {directory}")

except Exception as e:

print(f"Error creating directory {directory}: {e}")

raise


def read_tsv(file_path):

"""

Read a TSV file into a pandas DataFrame.

Parameters:

file_path (str): Path to the TSV file.

Returns:

DataFrame: The data read from the TSV file.

Raises:

Exception: If reading the file fails.

"""

try:

return pd.read_csv(file_path, sep='\t')

except Exception as e:

print(f"Error reading {file_path}: {e}")

raise


def write_tsv(data_frame, file_path):

"""

Write a pandas DataFrame to a TSV file.

Parameters:

data_frame (DataFrame): DataFrame to write.

file_path (str): Path to the output TSV file.

Raises:

Exception: If writing the file fails.

"""

try:

data_frame.to_csv(file_path, sep='\t', index=False)

print(f"Saved file: {file_path}")

except Exception as e:

print(f"Error writing {file_path}: {e}")

raise


def center_data(file_path, start_index, end_index):

"""

Center data by subtracting the mean of a specific range from all values.

Parameters:

file_path (str): Path to the TSV file.

start_index (int): Start marker index for centering.

end_index (int): End marker index for centering.

Returns:

DataFrame: The centered data.

"""

df = read_tsv(file_path)

marker = read_tsv(file_path.replace('.tsv', '_markers.tsv'))

start_frame = marker.iloc[start_index]['timestamp']

end_frame = marker.iloc[end_index]['timestamp']


mean_values = df.loc[(df['Frame'] >= start_frame) & (df['Frame'] <= end_frame)].mean()

centered_df = df.copy()

centered_df.loc[:, centered_df.columns != 'Frame'] -= mean_values


return centered_df


def smooth_array(array, gauss_sigma):

"""

Apply Gaussian smoothing to a numpy array.

Parameters:

array (numpy array): Input data to smooth.

gauss_sigma (float): Standard deviation for Gaussian kernel.

Returns:

numpy array: Smoothed data.

"""

return gaussian_filter1d(array, sigma=gauss_sigma, mode="nearest")

def smooth_laf(df, gauss_sigma_l=30, gauss_sigma_a=40, gauss_sigma_f=30):

"""

Apply Gaussian smoothing to specific columns of the DataFrame.

Parameters:

df (DataFrame): Input DataFrame with raw data.

gauss_sigma_l (float): Gaussian sigma for lateral bending.

gauss_sigma_a (float): Gaussian sigma for axial rotation.

gauss_sigma_f (float): Gaussian sigma for flexion/extension.

Returns:

DataFrame: The smoothed data.

"""


df['Smoothed_Lateral_Bending'] = smooth_array(df['T8_Head Lateral Bending'], gauss_sigma_l)

df['Smoothed_Axial_Rotation'] = smooth_array(df['T8_Head Axial Bending'], gauss_sigma_a)

df['Smoothed_Flexion/Extension'] = smooth_array(df['T8_Head Flexion/Extension'], gauss_sigma_f)

return df



# Statistical analysis

def statistics_real(input_file_path):

"""

Compute descriptive statistics for real (raw) smoothed data.

Parameters:

input_file_path (str): Path to the input TSV file.

Returns:

DataFrame: Descriptive statistics including min, max, mean, std, median, and quantiles.

"""

data = pd.read_csv(input_file_path, sep='\t')

marker_filename = input_file_path.replace('.tsv', '_markers.tsv')

marker = pd.read_csv(marker_filename, delimiter='\t')

start_frame = marker.iloc[1]['timestamp']


interval_data = data[(data['Frame'] >= start_frame)]

df = interval_data.copy()

statistics = {

'min': df[['Smoothed_Lateral_Bending', 'Smoothed_Axial_Rotation', 'Smoothed_Flexion/Extension']].min().tolist(),
```

```
'max': df[['Smoothed_Lateral_Bending', 'Smoothed_Axial_Rotation', 'Smoothed_Flexion/Extension']].max().tolist(),

'mean': df[['Smoothed_Lateral_Bending', 'Smoothed_Axial_Rotation', 'Smoothed_Flexion/Extension']].mean().tolist(),

'std': df[['Smoothed_Lateral_Bending', 'Smoothed_Axial_Rotation', 'Smoothed_Flexion/Extension']].std().tolist(),

'median': df[['Smoothed_Lateral_Bending', 'Smoothed_Axial_Rotation', 'Smoothed_Flexion/Extension']].median().tolist()
,

'25%qt': df[['Smoothed_Lateral_Bending', 'Smoothed_Axial_Rotation', 'Smoothed_Flexion/Extension']].quantile(0.25).t
olist(),

'75%qt': df[['Smoothed_Lateral_Bending', 'Smoothed_Axial_Rotation', 'Smoothed_Flexion/Extension']].quantile(0.75).t
olist()

}

return pd.DataFrame(statistics, index=['Smoothed_Lateral_Bending', 'Smoothed_Axial_Rotation', 'Smoothed_Flexion/Extension'])



def statistics_abs(input_file_path):

"""

Compute descriptive statistics for absolute values of smoothed data.

Parameters:

input_file_path (str): Path to the input TSV file.

Returns:

DataFrame: Descriptive statistics for absolute values of smoothed data.

"""

data = pd.read_csv(input_file_path, sep='\t')

marker_filename = input_file_path.replace('.tsv', '_markers.tsv')

marker = pd.read_csv(marker_filename, delimiter='\t')

start_frame = marker.iloc[1]['timestamp']



interval_data = data[(data['Frame'] >= start_frame)].copy()

interval_data[['Smoothed_Lateral_Bending', 'Smoothed_Axial_Rotation', 'Smoothed_Flexion/Extension']] = \

interval_data[['Smoothed_Lateral_Bending', 'Smoothed_Axial_Rotation', 'Smoothed_Flexion/Extension']].abs()



statistics = {

'min': interval_data[['Smoothed_Lateral_Bending', 'Smoothed_Axial_Rotation', 'Smoothed_Flexion/Extension']].min().tolist(),

'max': interval_data[['Smoothed_Lateral_Bending', 'Smoothed_Axial_Rotation', 'Smoothed_Flexion/Extension']].max().tolist(),

'mean': interval_data[['Smoothed_Lateral_Bending', 'Smoothed_Axial_Rotation', 'Smoothed_Flexion/Extension']].mean().tolist(),

'std': interval_data[['Smoothed_Lateral_Bending', 'Smoothed_Axial_Rotation', 'Smoothed_Flexion/Extension']].std().tolist(),

'median': interval_data[['Smoothed_Lateral_Bending', 'Smoothed_Axial_Rotation', 'Smoothed_Flexion/Extension']].median().tolist()
,

'25%qt': interval_data[['Smoothed_Lateral_Bending', 'Smoothed_Axial_Rotation', 'Smoothed_Flexion/Extension']].quantile(0.25).t
olist(),

'75%qt': interval_data[['Smoothed_Lateral_Bending', 'Smoothed_Axial_Rotation', 'Smoothed_Flexion/Extension']].quantile(0.75).t
olist()

}

return pd.DataFrame(statistics, index=['Smoothed_Lateral_Bending', 'Smoothed_Axial_Rotation', 'Smoothed_Flexion/Extension'])



def get_symmetric_y_range(data_list):

"""Calculate symmetric y-axis limits based on the largest absolute min/max value across all centered data."""

global_min = min(df.iloc[:, 1:].min().min() for df in data_list)

global_max = max(df.iloc[:, 1:].max().max() for df in data_list)



# Determine the larger absolute value and set symmetric range

max_abs_value = max(abs(global_min), abs(global_max))

y_min, y_max = -max_abs_value, max_abs_value

return y_min, y_max



def plot_data(df, marker, file_path, output_plot_directory, y_min, y_max, plot_type="all"):

"""

Generate plots with fixed y-axis limits.

Parameters:

df (DataFrame): The DataFrame containing data to plot.

marker (DataFrame): The marker DataFrame or None.

file_path (str): The path of the file being processed.

output_plot_directory (str): Directory to save the plot.

y_min (float): Minimum y-axis value.

y_max (float): Maximum y-axis value.

plot_type (str): Type of columns to plot ('transformed' or 'smoothed').

"""

plt.figure(figsize=(10, 6))



# Determine which columns to plot

if plot_type == "transformed":

columns_to_plot = [col for col in df.columns if col not in ['Frame']]

elif plot_type == "smoothed":

columns_to_plot = [col for col in df.columns if col.startswith("Smoothed_")]

else:

columns_to_plot = df.columns



# Plot the selected columns

for column in columns_to_plot:

if column != 'Frame':

plt.plot(df['Frame'], df[column], label=column)



# Plot markers if available

if marker is not None:

marker_timestamps = marker['timestamp'].values

for timestamp in marker_timestamps:

plt.axvline(x=timestamp, color='r', linestyle='--')



plt.xlabel('Frame')

plt.ylabel('Value')

plt.ylim(y_min, y_max)

plt.title(os.path.splitext(os.path.basename(file_p
ath))[0])

plt.legend()

plt.grid(True)



# Adjust plot filename based on type

if plot_type == "transformed":

plot_filename = os.path.splitext(os.path.basename(file_path))[0] + '_transformed_plot.png'

elif plot_type == "smoothed":

plot_filename = os.path.splitext(os.path.basename(file_path))[0] + '_smoothed_plot.png'

else:

plot_filename = os.path.splitext(os.path.basename(file_path))[0] + '_plot.png'



plot_filepath = os.path.join(output_plot_directory, plot_filename)

plt.savefig(plot_filepath)

plt.close()
```

```
print(f"Plot saved successfully: {plot_filepath}")

def main():

# Check if the script is running in the correct Conda environment

check_environment(expected_env='rescue')

parser = argparse.ArgumentParser(

description="""

Process data files for transformation, smoothing, and statistical analysis.

Usage Examples:

---------------

1. Convert Excel to TSV:

python Hill.py --mode excel_to_tsv

2. Transform Data:

python Hill.py --mode transform -i "path/to/input" -o "path/to/output" --start 0 --end 10

3. Smooth Data:

python Hill.py --mode smooth -i "path/to/transformed/data" -o "path/to/smoothed/output"

4. Generate Statistics:

python Hill.py --mode stats -i "path/to/smoothed/data" -o "path/to/statistics/output"

""",

formatter_class=argparse.RawTextHelpFormatter

)

parser.add_argument("--mode", type=str, required=True, choices=["excel_to_tsv", "transform", "smooth", "stats"],

help="Mode to run the script in: excel_to_tsv, transform, smooth, or stats.")

parser.add_argument("-i", "--input_directory", type=str, required=True, help="Input directory containing TSV files")

parser.add_argument("-o", "--output_directory", type=str, required=True, help="Output directory for processed files")

parser.add_argument("--start", type=int, help="Start index for processing (only for transform mode)")

parser.add_argument("--end", type=int, help="End index for processing (only for transform mode)")

args = parser.parse_args()

ensure_directory_exists(args.output_directory)

if args.mode == "excel_to_tsv":

excel_to_tsv(input_folder=args.input_directory, output_folder=args.output_directory)

elif args.mode == "transform":

# Create plots directory only for transform mode

output_plot_directory = os.path.join(args.output_directory, "plots")

ensure_directory_exists(output_plot_directory)

centered_data_list = []

file_paths = []

for filename in os.listdir(args.input_directory):

if filename.endswith('.tsv') and "markers" not in filename:

file_path = os.path.join(args.input_directory, filename)

centered_df = center_data(file_path, args.start, args.end)

centered_data_list.append(centered_df)

file_paths.append(file_path)

output_file_path = os.path.join(args.output_directory, filename)

write_tsv(centered_df, output_file_path)

# Copy the marker file to the output directory

marker_file = file_path.replace('.tsv', '_markers.tsv')

if os.path.exists(marker_file):

output_marker_file = os.path.join(args.output_directory, os.path.basename(marker_file))

shutil.copy(marker_file, output_marker_file)

print(f"Copied marker file: {output_marker_file}")

y_min, y_max = get_symmetric_y_range(centered_data_list)

for file_path, centered_df in zip(file_paths, centered_data_list):

marker_path = file_path.replace('.tsv', '_markers.tsv')

marker = read_tsv(marker_path)

plot_data(centered_df, marker, file_path, output_plot_directory, y_min, y_max)

elif args.mode == "smooth":

# Create plots directory only for smooth mode

output_plot_directory = os.path.join(args.output_directory, "plots")

ensure_directory_exists(output_plot_directory)

smoothed_data_list = []

file_paths = []

for filename in os.listdir(args.input_directory):

if filename.endswith('.tsv') and "markers" not in filename:

file_path = os.path.join(args.input_directory, filename)

df = read_tsv(file_path)

smoothed_df = smooth_laf(df)

smoothed_data_list.append(smoothed_df)

file_paths.append(file_path)

output_file_path = os.path.join(args.output_directory, filename)

write_tsv(smoothed_df, output_file_path)

# Copy the marker file to the output directory

marker_file = file_path.replace('.tsv', '_markers.tsv')

if os.path.exists(marker_file):

output_marker_file = os.path.join(args.output_directory, os.path.basename(marker_file))

shutil.copy(marker_file, output_marker_file)

print(f"Copied marker file: {output_marker_file}")

y_min, y_max = get_symmetric_y_range(smoothed_data_list)

for file_path, smoothed_df in zip(file_paths, smoothed_data_list):

marker_path = file_path.replace('.tsv', '_markers.tsv')

marker = read_tsv(marker_path)

plot_data(smoothed_df, marker, file_path, output_plot_directory, y_min, y_max, plot_type="smoothed")

elif args.mode == "stats":

for filename in os.listdir(args.input_directory):

if filename.endswith('.tsv') and "markers" not in filename:

file_path = os.path.join(args.input_directory, filename)

stats_real = statistics_real(file_path)

stats_abs = statistics_abs(file_path)

output_file_path_real = os.path.join(args.output_directory, f"{os.path.splitext(filename)[0]}_stats_real.xlsx")

output_file_path_abs = os.path.join(args.output_directory, f"{os.path.splitext(filename)[0]}_stats_abs.xlsx")

stats_real.to_excel(output_file_path_real)

stats_abs.to_excel(output_file_path_abs)

if __name__ == "__main__":

main()
```
