# Supplementary material for: Effectiveness and safety of techniques for cervical spine immobilization in mountain rescue
Source: Scand J Trauma Resusc Emerg Med. 2026 Jan 16;34:12. doi: 10.1186/s13049-025-01530-z (PMC12849128; doi:10.1186/s13049-025-01530-z)
Supplement: Supplementary file 5 — Supplementary Material 5. Figure S1: Comparative plots without (a) and with (b) smoothing. The x-axis shows the measurement times (frames), recorded at a frequency of 240 per second [Hz], while the y-axis shows the angle changes [°]. Figure S2: Schematic overview of the evaluation steps. Figure S3: Proposal for a possible procedure for patient care with regard to cervical spine immobilization in mountain rescue based on the findings of this study. Table S1: Placement of the sensors [21]. Table S2: Anthropometric data of the test subject [21]. [file 13049_2025_1530_MOESM5_ESM.docx]

## Figures

Figure S 1: Comparative plots without (a) and with (b) smoothing. The x-axis shows the measurement times (frames), recorded at a frequency of 240 per second [Hz], while the y-axis shows the angle changes [°].


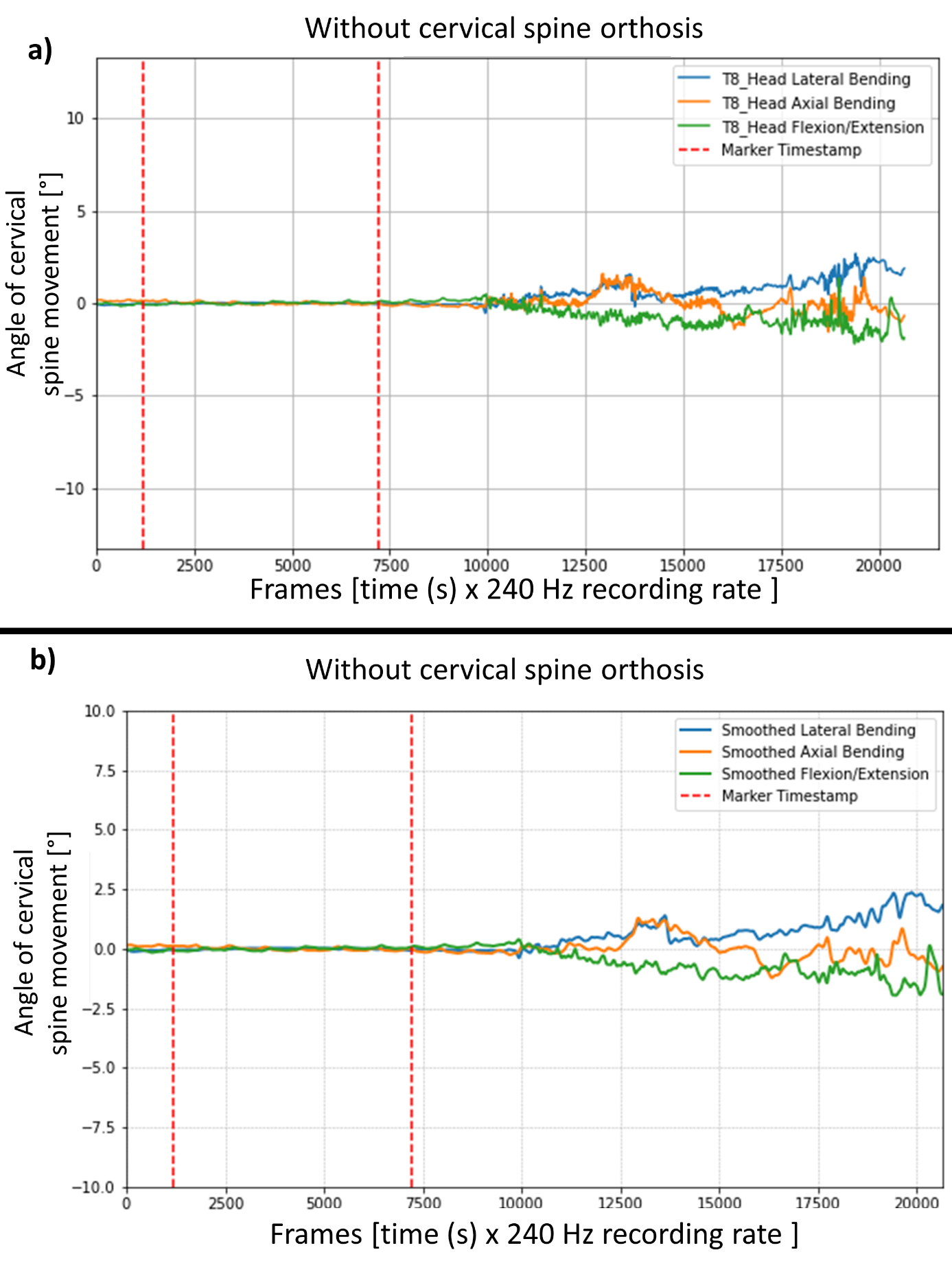


Figure S 2: Schematic overview of the evaluation steps


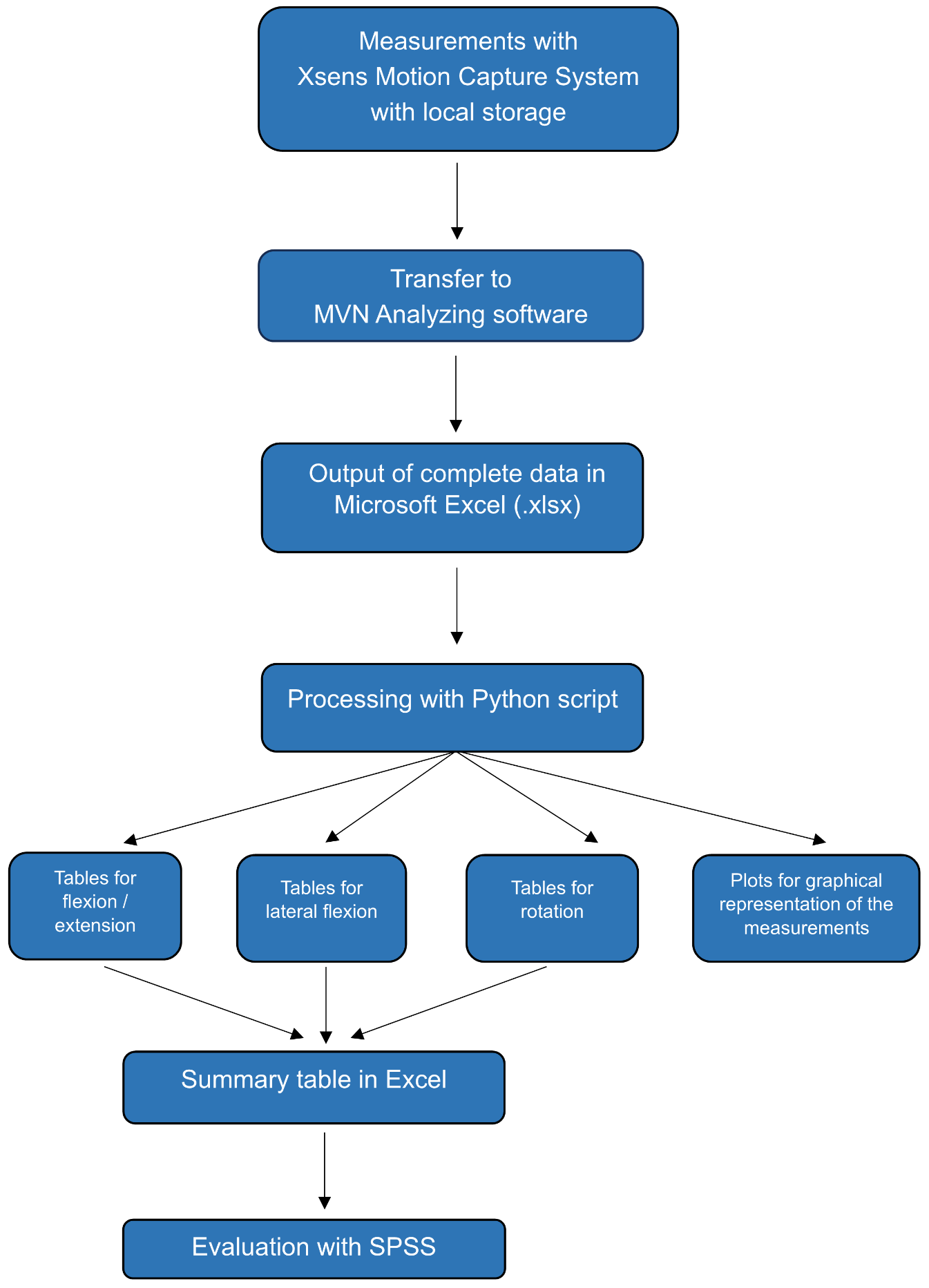


Figure S 3: Proposal for a possible procedure for patient care with regard to cervical spine

immobilization in mountain rescue based on the findings of this study


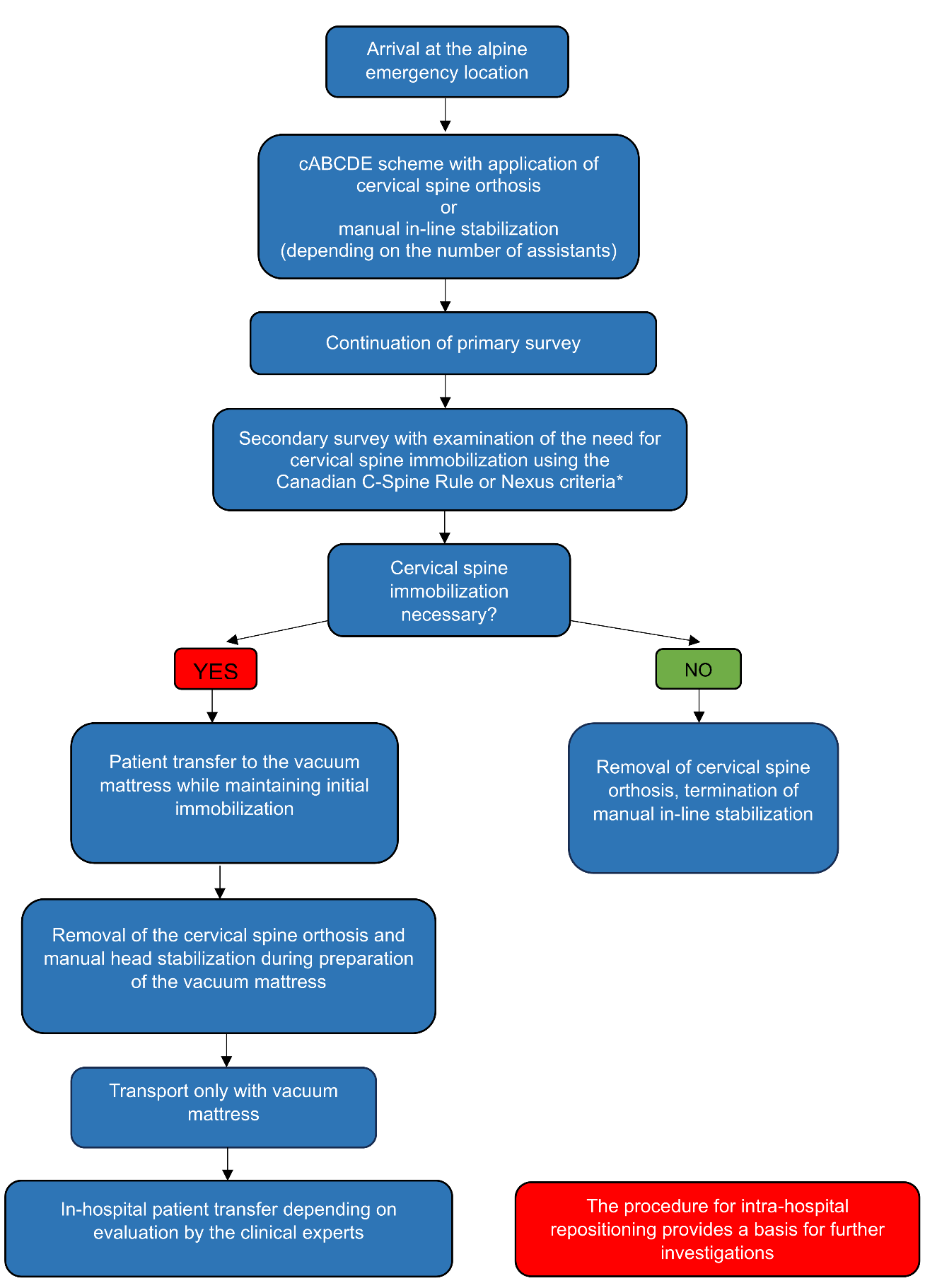


** Note: Additional secondary survey methods exist (such as those recommended by Cowley et al. [12]). However, these approaches have not yet been validated.*

## Tables

Table S 1: Placement of the sensors [21]

| Body region | Designation in the system | Optimum position |
| --- | --- | --- |
| Foot | FOOT | dorsum of foot, centered |
| Lower leg | iLEG | medial surface of tibia |
| Upper leg | uLEG | above the knee, lateral |
| Pelvis | PELV | sacral bone |
| Sternum | STERN | centered on the thorax |
| Shoulder | SHOU | scapula |
| Upper arm | uARM | above the elbow, lateral |
| Forearm | fARM | above the wrist, lateral |
| Hand | HAND | dorsum of hand |
| Head | HEAD | comfortable position for the test subject; in our measurements: forehead, centered |

Table S 2: Anthropometric data of the test subject [21]

| Dimensions | Description |
| --- | --- |
| Body height | Ground to top of head when standing upright |
| Foot/shoe length | Length of feet or shoes |
| Shoulder height | Ground to C7 spinous process |
| Shoulder width | Distance between left and right tip of acromion |
| Elbow span | Distance between elbows in T-pose (standing upright with both arms abducted to 90°) |
| Wrist span | Distance between wrists in T-pose |
| Arm span | Distance between fingertips in T-pose |
| Pelvic height | Ground to greater trochanter |
| Pelvic width | Distance between left and right anterior superior iliac spine |
| Knee height | Ground to lateral femoral epicondyle |
| Ankle height | Ground to tip of lateral malleolus |
