# Supplementary material for: Effectiveness and safety of techniques for cervical spine immobilization in mountain rescue
Source: Scand J Trauma Resusc Emerg Med. 2026 Jan 16;34:12. doi: 10.1186/s13049-025-01530-z (PMC12849128; doi:10.1186/s13049-025-01530-z)
Supplement: Supplementary file 4 [file 13049_2025_1530_MOESM6_ESM.docx]

Supplement

Section S1

Statistical Methods

The analysis software SPSS® Statistics (Version 29.0.0.0 (241), IBM, Armonk, USA) was used for statistical evaluation. All captured and already calculated values from the individual Excel files were combined into an overall table and divided into two groups. Group 0 included the runs without a cervical spine orthosis, and Group 1 the runs with a cervical spine orthosis. The table was then imported into the statistics program. Initially, a test for normal distribution was performed for all relevant groups by means of the Kolmogorow-Smirnow test. An exploratory data analysis was carried out during which the mean values (MV) as well as the standard deviation (± SD) and standard errors (SEM) were calculated. The median values and interquartile ranges (IQR) were also determined. A subsequent post hoc analysis was performed using the Mann–Whitney U test. An error probability of p < 0.05 was considered statistically significant.

Section S2

Evaluation of the measured results

A variety of factors had to be taken into account when choosing a suitable terrain for conducting the measurements. Our objective was to simulate the loads acting on the patient during transportation as realistically as possible. After careful analysis of potential rescue scenarios, the decision was made to simulate the rescue of an accident victim with a mountain stretcher. The route chosen for the measurements should have a significant difference in altitude as well as narrow bends and obstacles that are typical of alpine terrain. The testing area met these requirements and offered ideal conditions with different types of ground surfaces (gravel, forest ground, and meadow), sharp bends, and a 1.7-m-high hedge. To recreate real conditions, a square piece of timber was used to simulate the passing over a large root or stone, which often occurs in mountain rescue operations. The simulated (and therefore relatively short) rescue measures were performed by active members of the Bavarian Mountain Rescue Service, who were instructed and trained in how to handle the used materials. The Stifneck® Select™ cervical spine orthosis was applied according to the manufacturer’s instructions [17]. To guarantee efficacy, a series of measures was implemented to ensure the reproducibility of the test measurement runs. The measurement route was meticulously delineated and marked with signal cones. Initially, the WiFi network that had been configured by the author was found to be incompatible with transmitting stable data from the measurement system to the laptop. Therefore, the data was saved locally in an "on-body recording" mode. The values were then transmitted via a wired connection. In contrast to the measurements taken with a test person in our study, Grenier et al. used a dummy with an integrated measurement system in their study on immobilizing victims of skiing accident [26]. However, the technology of the Xsens motion capture system described under 2.3 allowed detailed and precise recordings. The effectiveness of this system had already been proven in several studies, including the aforementioned studies by Morag et al. [23], Kieninger et al. [24], and Uzun et al. [25], which provided excellent results.

The recorded values were processed according to the methods described under Sect. "Processing of the measurements" in order to be able to make reliable and reproducible statements. By analyzing the graphical representation of the measured data, deviations in the time course of the transport could be identified, and the time markers were adjusted accordingly. Thus, a precise analysis of the movements during the measurements was ensured, excluding potential erroneous influences during the determination of the resting position and subsequent to transportation. The parameters for the Gaussian smoothing filter were defined for subsequent smoothing of the data. The objective here was to sufficiently reduce the displayed noise signals without deleting actually relevant and recorded movements. The measuring tolerance of 1° specified by the manufacturer of the recording system was used as a guide [21]. After analyzing the individual axes in the raw plots (see Figure S 1), the kernel sizes were defined in such a way that erroneous signals were smoothed to significantly less than 1°.

Since the noise in the individual planes of movement was more or less strongly pronounced, the kernel sizes were adjusted individually for each axis. This adjustment resulted in values of 30 for flexion/extension as well as lateral flexion, and of 40 for rotation. The kernel sizes describe the standard deviation (sigma) of the Gaussian smoothing filter and thus the strength of smoothing. As described in Sect. "Statistics", the statistical evaluation was carried out with the assistance of the Methods Consulting Service of the University of Zurich. The Kolmogorow-Smirnow tests showed a normal distribution for the rotation data so that an evaluation by means of t-tests would have been possible in principle. However, given the fact that there were only 15 test runs per group, this sample size appeared to be too small to be able to make a reliable statement on normal distribution. Therefore, it was decided to use the Mann–Whitney U test for all variables in both groups.

Section S3

Development of Immobilization Materials

Due to economic pressure and new findings, medical technology companies regularly develop new products. Thus, a myriad of different variants of vacuum mattresses and rigid cervical spine orthoses are used in prehospital emergency care. Thanks to the close cooperation with the Bavarian Mountain Rescue Service, this study relied on the equipment generally used by rescuers to provide them with familiar tools and to ensure the realistic execution of the simulated operations. The “80 – Bayern” type vacuum mattress is currently widely used by mountain rescue teams, but it has gradually been replaced by a new model since 2024. The design of the new model is similar to that of the “Schnitzler 816 K-TV-H + ” type mattress (Schnitzler Rettungsprodukte GmbH & Co. KG, Niederkassel-Mondor, Germany) already used by the Bavarian Rescue Service. This product was also used in the above-mentioned study by Kieninger et al. [24]. Unlike the “80 – Bayern” type mattress, newer models can be adjusted to fit the head particularly well and are secured with forehead and chin straps to provide better stabilization of the head during immobilization using only a vacuum mattress. Especially in the sagittal plane, the additional straps may reduce increased flexion values (described in Sect. "Processing of the measurements". and Table 3) during transportation without a cervical spine orthosis. Also, the procurement of a new mountain stretcher is underway. The “Steelight” stretcher is already being replaced by the successor model “Tyral”, also from Tyromont. Both products are based on a comparable design principle and serve exclusively as a means of transport, not immobilization. Therefore, use of the older “Steelight” stretcher seems to have no influence on the measurement results. There is also a wide range of rigid cervical spine orthoses to choose from. Nolte et al. used an Ambu® Perfit ACE type rigid cervical spine orthosis (Ambu GmbH, Bad Nauheim, Germany) in their study on cervical spine immobilization during patient transportation in an ambulance [27]. This orthosis is widely used in Germany and, according to Jung et al., provides stable immobilization results. The differences in the remaining range of motion in inclination and lateral flexion between Ambu® Perfit ACE and Laerdal Stifneck® were not significant [28].

When planning our study, we originally considered to also investigate the effectiveness of soft cervical spine orthoses. In Germany, rigid cervical spine orthoses are used in prehospital emergency medicine including mountain rescue. When investigating the neurological outcome of patients whose cervical spines were immobilized with soft cervical spine orthoses in the emergency room, Asha et al. found that these do not increase the risk of secondary spinal cord damage [29]. When investigating the pain perception and acceptance of cervical spine orthoses, Baker et al. found that patients fitted with soft cervical spine orthoses experience less agitation and pain [30]. Since both the Bavarian ground-based and mountain rescue services exclusively use rigid cervical spine orthoses and do not plan to switch to or procure soft cervical spine orthoses, it was decided that measurements would be taken with the materials currently used in emergency care.

Section S4

Limitations

The simulated rescue conditions, although designed to be as realistic as possible, may not entirely encompass the unpredictability and variability inherent in actual mountain rescue scenarios. Real-life situations often involve unforeseen challenges, changing weather, and complex terrain, which can significantly impact the rescue operation and its outcomes. It is important to acknowledge that not all the factors under consideration could be fully replicated in our simulation.

The study design has several limitations. Most notably, the use of one male test person limits the generalizability of the findings. The measurements were carried out on a person who was awake, responsive, and had preserved protective reflexes. Therefore, we cannot provide data or insights related to variables, such as body mass index, stature, or differences in the anatomy of men, women, and children. Furthermore, stabilization of the head by involuntary muscle contraction could not be excluded in our experiments. While the study design allowed for controlled and detailed data collection, further research involving a more diverse group of test persons is necessary to validate and expand upon these results.
